# The challenge of managing the evolution of genomics data over time: a conceptual model-based approach

**DOI:** 10.1186/s12859-022-04944-z

**Published:** 2022-11-09

**Authors:** Alberto García S., Mireia Costa, Ana Leon, Oscar Pastor

**Affiliations:** PROS Research Center, VRAIN Research Institute, Universitat Politècnica de València, Camino de Vera, Valencia, Spain

**Keywords:** Bioinformatics, Temporal dimension, Conceptual models, SILE method, DELFOS oracle

## Abstract

**Background:**

Precision medicine is a promising approach that has revolutionized disease prevention and individualized treatment. The DELFOS oracle is a model-driven genomics platform that aids clinicians in identifying relevant variations that are associated with diseases. In its previous version, the DELFOS oracle did not consider the high degree of variability of genomics data over time. However, changes in genomics data have had a profound impact on clinicians’ work and pose the need for changing past, present, and future clinical actions. Therefore, our objective in this work is to consider changes in genomics data over time in the DELFOS oracle.

**Methods:**

Our objective has been achieved through three steps. First, we studied the characteristics of each database from which the DELFOS oracle extracts data. Second, we characterized which genomics concepts of the conceptual schema that supports the DELFOS oracle change over time. Third, we updated the DELFOS Oracle so that it can manage the temporal dimension. To validate our approach, we carried out a use case to illustrate how the new version of the DELFOS oracle handles the temporal dimension.

**Results:**

Three events can change genomics data, namely, the addition of a new variation, the addition of a new link between a variation and a phenotype, and the update of a link between a variation and a phenotype. These events have been linked to the entities of the conceptual model that are affected by them. Finally, a new version of the DELFOS oracle that can deal with the temporal dimension has been implemented.

**Conclusion:**

Huge amounts of genomics data that is associated with diseases change over time, impacting patients’ diagnosis and treatment. Including this information in the DELFOS oracle added an extra layer of complexity, but using a model-driven based approach mitigated the cost of implementing the needed changes. The new version handles the temporal dimension appropriately and eases clinicians’ work.

## Background

The complexity of the domain under study and the particularities of our use case require us to provide as much context as possible. We begin this section by introducing the basics of precision medicine and the current problems that arise from poor data management strategies. Then, we present the SILE method, which defines four stages to systematically identify relevant variations for a given disease with appropriate data quality and data-filtering standards. SILE requires proper ontological support to achieve its purposes. The following sections describe the two most common approaches for providing ontological support, namely, genomics ontologies and conceptual models. We decided to use conceptual modeling techniques to generate the Conceptual Schema of the Human Genome (CSHG) and adapt it to the particularities of our use case. This section ends with a detailed description of the DELFOS Oracle platform, which is the technological implementation of the SILE method that is guided by our conceptual schema.

### Introduction

The practice of medicine is currently undergoing a paradigm shift towards disease prevention and individualized treatment, where *precision medicine* has emerged as a promising approach for achieving these goals. According to [1], precision medicine is defined as a computational approach that integrates and interprets *omics* data to treat every patient as an individual case. Omics is a general term that is used to encompass several sub-terms. The addition of the “omics” prefix to a molecular term implies a comprehensive assessment of a set of molecules [2]. One of the omics with the most impact in precision medicine is Genomics, which focuses on the study of the genome from multiple perspectives. For instance, one of the applications of Genomics is identifying genetic variations that are associated with diseases or response to treatments.

In precision medicine, identifying relevant variations associated with diseases with high-quality evidence is essential for efficient prevention, diagnosis, and treatment. This process should use several databases to ensure completeness. However, clinicians struggle when working with them. The reasons for this situation are complex and diverse [3, 4], but we can highlight the following as being the most relevant ones:

- The huge volume of publicly available data [3, 5]: Data acquisition is highly distributed, involving several heterogeneous formats. The growth rate of genomic data is astonishing, and it is expected to double every 7-12 months. Also, the number of existing databases is enormous. As of this writing, there are 1,641 databases, and dozens of them are created or become obsolete every year.
- The complexity of integrating omics data [6]: Because of the huge volume of publicly available data that is stored in different formats, the amount of effort required to integrate it is considerable. Several data transformation and data integration tasks, such as entity and attribute matching, remain highly manual.
- The complexity of dealing with the quality issues that are inherent to genomic data [7]: Although genomics analyses are highly dependent on the quality of the data used, data quality processes have not been given much attention. Redundant and non-curated data coexists with highly accurate data that has been manually reviewed.
- The high degree of variability of genomic data over time [8]. Genomic data is continuously changing, ranging from the set of genes considered of interest for a disease to even the sequence of reference used to perform variant calling. Here, the reclassification of variation pathogenicity over time requires special attention because of its implications at the clinical level [9]. Our knowledge regarding variation pathogenicity is dynamic and constantly growing, making it necessary to define standardized processes to keep the information updated.

This manuscript focuses on how to manage the high degree of variability of genomic data over time. This data is stored in several databases, some of which contain variations that are linked to a wide range of phenotypes (e.g., ClinVar [10], Ensembl [11], or GWAS Catalog [12]), while othe databases focus on a specific disease (e.g., Cosmic [13] or CardioDB [14]).

The high variability over time of the information that is associated with genetic variations has significant consequences and complicates achieving high-quality precision medicine. For instance, variant classification is an essential task in this context, and a high rate of variation reclassification can be a barrier to achieving high-quality precision medicine. Although little of the published data quantifies the prevalence of this reclassification, the existing evidence indicates that variation reclassification over time is not a rare event [15]. Variation reclassification can have dramatic consequences depending on the affected disease. For instance, variation reclassification is commonplace in hereditary cancer predisposition testing [16], which has a psychosocial impact on individuals and affects family communication [17]. Another example is found in the diagnosis of inherited arrhythmogenic syndromes, where 70% of rare genetic variations associated with these syndromes have been reclassified over time [18]. Besides variations, the significance of other genomic components, like genes, changes over time. For instance, while the American College of Medical Genetics (ACMG) recommended conducting genomics studies on a set of 56 genes in 2013, this list was expanded with 13 additional genes in 2021 [19].

This situation is clearly visible in the case of one of the most popular genomic databases, ClinVar. The ClinVar database updates the relationship between variations and disease development every week (''Study the characteristics of each database from which the DELFOS oracle extracts data'' section). Due to its high update rate, it is a perfect candidate for studying the impact of the changes in the pathogenicity of variations and their associated information over time.

**Fig. 1:**
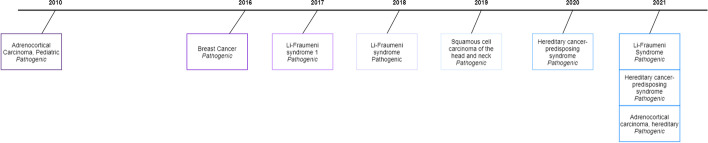
Changes over time of the variation *NC_000017.11:7670698:C:T*

The *NC_000017.11:7670698:C:T*^1^,^2^ variation is an excellent example of how new evidence associating a variation with a new phenotype appears over time. Figure 1 presents how the relations of this particular variation with different phenotypes evolve over time.

The first variation-phenotype link for this variation was reported for *Adrenocortical Carcinoma, Pediatric* in 2010, and no new information was added until 2016. From that moment, new links associating the variation with additional phenotypes appear every year. It is worth mentioning that three new links with new phenotypes (*Li-Fraumeni Syndrome, Hereditary cancer-predisposing syndrome, Adrenocortical carcinoma hereditary*) appeared in just one year (2021), which suggests that the temporality of the genomic information is gaining relevance over time.

**Fig. 2:**
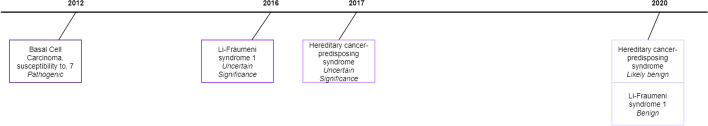
Changes over time of the variation *NC_000017.11:7668433:T:G*

Another example is the *NC_000017.11:7668433:T:G*^3^ variation. The study of this variation allows us to illustrate how new evidence about a previously reported association appears over time. Figure 2 presents how the relations of this particular variation evolve over time.

In this case, we see that, in 2016 and 2017, there were reported relationships with the phenotypes *Li-Fraumeni syndrome 1* and *Hereditary cancer-predisposing syndrome*. In both cases, it was determined that the variation had an uncertain significance. However, advancing in time to the year 2020, two new relations for the same phenotype determined that the variation should be classified as Likely benign/benign. In this case, the evolution of the knowledge about these phenotypes leads to a reevaluation of the variations’ pathogenicity.

The examples provided demonstrate the importance of studying the variability of the genomic variation’s information over time, as it could have consequences in the decisions taken in clinical care.

Since addressing the management of the high degree of variability of genomic data over time in its entire dimension is not feasible, we have focused on a specific domain in order to create a first approach that can be generalizable to other areas. This domain is *the application of the SILE Method, which was designed for the management of genomic data (see * ''The SILE method'' section), *for precision medicine.* Specifically, this work focuses on incorporating the management of the variability of genomic data in the DELFOS Oracle (see ''The DELFOS oracle'' section), which is a platform that implements the SILE method in the specific task of identifying the DNA variations that are relevant enough to be used in a clinical context.

### The SILE method

The SILE method is a method that provides a systematic way to deal with the problems that arise in the variation-curation process, i.e., how to deal with huge amounts of data, the heterogeneity of the data and the quality of the data [20]. This method is divided into four phases that are applied consecutively:

1. *The Search phase (S)* This phase consists of selecting the relevant (and appropriate) genomics repositories based on requirements defined previously. Once the repositories are selected, the data stored in them must be extracted in a processable format so that it can be analyzed in the next phase.
2. *The Identification phase (I)* This phase consists of the identification of high-quality data from among all the data extracted in the Search phase by using Artificial Intelligence algorithms.
3. *The Load phase (L)* This phase consists of the definition and implementation of the loading process to store the data that is identified as relevant in the Identification phase.
4. *The Exploitation phase (E)* This phase consists of exploiting the data stored in the Load phase in a clinical context to generate valuable knowledge.

One of the main characteristics of the SILE method is that it is model-based. This means that the whole process is ontologically grounded on a Conceptual Schema (CS). This feature is not common in other genomics methods, and few of the existing works can be considered a pure conceptual modeling approach, although there are some exceptions [21–24]. The use of genomics ontologies is more common, and they are used for defining relevant concepts and their relationships in genomics.

### Genomics ontologies

As mentioned above, most of the existing work consists of the definition of genomics ontologies, for instance, the Open Biological and Biomedical Ontology (OBO) Foundry [25], which is an entity whose mission is to provide a set of design ontology principles. As a result, hundreds of ontologies have been defined in an attempt to follow the OBO principles. The main benefit of using genomics ontologies is that they help in reducing domain heterogeneity by providing well-defined standards for some specific concepts of the domain. Ontologies also allows for standard data concepts that capture the semantics of clinical information to be defined.

However, using such ontologies may have undesired consequences [26] because they have inherent ontological weaknesses [27] and they are hard to maintain [28]. Besides, ontologies alone are not sufficient for exchanging information among systems. Conceptualization techniques are required since the definition of domain terms relies on human experience, with abstract and ambiguous definitions. The use of genomics ontologies is an appropriate approach for solving some of the existing problems, but they are not enough to solve all of them. Therefore, the use of conceptual modeling (CM) techniques that complement the use of genomics ontologies can help in defining ambiguous terms and the relationships among them.

### Conceptual modeling

Conceptual modeling is defined as the activity of describing aspects of the world for the purpose of understanding and communication. It answers fundamental questions by identifying what concepts are relevant and their relationships with each other, which helps to establish common ontological frameworks. The use of CM has additional benefits. On the one hand, translating biological definitions into digital artifacts that can be used by software and programs is required, and CM can ease the translation of biological information into computational concepts. A correct computational representation of biological concepts leads to an increase in computational performance, a decrease in misunderstandings, and better manipulation and data transformation capabilities. On the other hand, CM can help in making genomic data FAIR-compliant [29]. In fact, semantic interoperability cannot be achieved without proper conceptualization [30].

The use of CM has recently been gaining attention in genomics. The Global Alliance for Genomics and Health^4^ is committed to using models for creating standards that improve the interoperability and sharing of genomic data; the Data-driven Genomic Computing (GeCo) project uses CM to describe semantically heterogeneous data and providing data interoperability [21]; and CM has been successfully used to support virology research in the context of the COVID pandemic outbreak [22].

### The conceptual schema of the genome

As stated above, within the framework of this research, the use of CM is a central part of the SILE method. This method uses the Conceptual Schema of the Genome (CSG) [31, 32] as a basis for its ontological background. This conceptual schema seeks to be the single point of truth when working with genomic data. It also provides a holistic perspective by characterizing the main entities between the genotype and its corresponding phenotype. The CSG is divided into six different views:

- The structural view, which describes the different components that characterize the structure of the human genome.
- The variation view, which identifies and characterizes changes in the sequence of the genome.
- The transcription view, which models the transcription process, including both the synthesis of proteins and non-coding RNAs.
- The protein view, which characterizes the different structures of proteins, including their physical and chemical characteristics.
- The pathway view, which represents the set of metabolic pathways of organisms.
- The phenotype view, which defines the most relevant concepts regarding the phenotypes that are expressed in our bodies and the link between them and variations.
- The bibliography and databank view, which details external information and sources related to the elements of the conceptual schema.

The CSG provides a holistic perspective of the genome; however, the number of concepts and relationships that are represented can be too many for the needs of most real-world use cases. For instance, comparative DNA studies will hardly use any concepts of the protein view. Therefore, we need a systematic way to reduce the number of concepts that we represent, which can reduce the complexity of the data and facilitate their management. To deal with this situation, we have created the ISGE method [33].

### The ISGE method and the conceptual schema of the SILE method

The ISGE is used to generate “conceptual views” of the CSG that are tailored to the specific needs of the use cases.

**Fig. 3:**
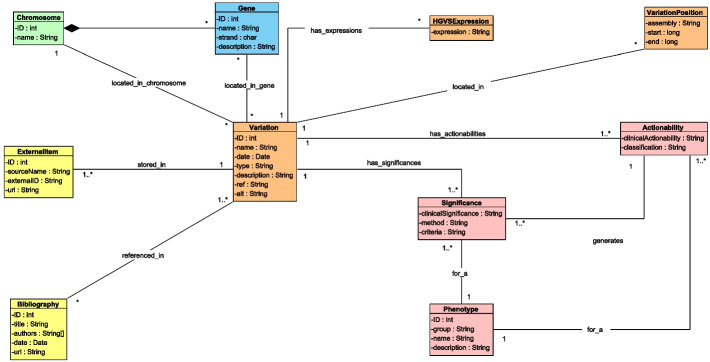
UML class diagram representing the Conceptual Schema that is the foundation of the SILE Method (CSSM). The structural view is depicted in green. The variation view is depicted in orange. The transcription view is depicted in blue. The bibliography and databank view is depicted in yellow. The phenotype view is depicted in pink

We have used the ISGE method to create a conceptual view of the CSG, focusing on the specific requirements of the SILE method. We called this conceptual view the Conceptual Schema of the SILE Method (CSSM), which is shown in Fig. 3.

The central entity of the CSSM is the Variation, which represents a change in the DNA sequence and is identified by an ID, a name, and a description. A Variation is defined by the reference and the alternative alleles, (i.e., the change produced in the genome sequence), the variation type, and the last time it was analyzed by the SILE method. variations can be complemented with a set of HGVSExpressions^5^. variations are referenced in at least one of the external databases used by the SILE method. These databases are represented in the conceptual schema with the ExternalItem class. An ExternalItem is characterized by the name of the database located in (source), the URL to access it, and the variation’s specific identifier in the data source.

There are several DNA sequences in our Chromosomes with specific functionality. In this use case, we only focus on Genes, which are characterized by an internal ID, its official name, its direction in the DNA (strand), and a description. The location of a variation is represented in three ways (from more general to more specific): i) every Variation is located in exactly one Chromosome; ii) every variation can be located in a set of Genes; and iii) every variation can be located in a specific position in the complete DNA sequence, through the VariationPosition class. It is important to note that the exact position of a Variation can be unknown, in which case the Variation is considered to be imprecise. Besides, the position can change depending on the specific assembly (an attribute of the VariationPosition class) that is used to locate the Variation.

Variations are linked to Phenotypes. In this model, a phenotype represents a disease. Each Variation-Phenotype pair is connected through a set of clinical Significances that represents the pathogenicity (i.e. pathogenic or benign) of the Variation with regard to the Phenotype. The different Significances are provided by researchers, who submit the results of their research along with the specific method and criteria that they followed. Additionally, these Significances are aggregated in a computed value that is represented through the Actionability class [34]. This class is an aggregated value of the significances that represents: i) the practical importance of the variation for a phenotype (clinicalActionability attribute); and ii) the classification (classification attribute), which represents the quality of the evidence that supports such Actionability (i.e., strong evidence, moderate evidence, limited evidence, or to follow up).

Each variation can contain Phenotype-related Bibliography. This information is stored using the Bibliography concept, which is characterized by a title, the list of authors, the publication date, and a URL to access it.

So far, we have described the SILE method and the conceptual schema that supports its ontological background. As mentioned above, the SILE method provides a systematic way to deal with the problems that arise in the variation-curation process. We have created a genomics platform that provides support to the four phases of the SILE method, called the DELFOS Oracle.

### The DELFOS oracle

The SILE method is a generic method that provides solutions to problems that are related to genomic data management. While the method provides a set of guidelines to follow, the particularities of each use case guide the specific instantiations of SILE.

One such instantiation consists of the identification of DNA variations that are relevant for use in clinical practice [35], which typically requires great amounts of manual effort. The DELFOS oracle is a plataform that facilitates this specific instantiation by automating most of the aforementioned manual tasks. This instantiation is an attractive and relevant case of study in a promising and challenging context, i.e., diagnosis and treatment selection in personalized precision medicine. More details regarding the relevance of this task can be found in ''A real-world need'' section.

**Fig. 4:**
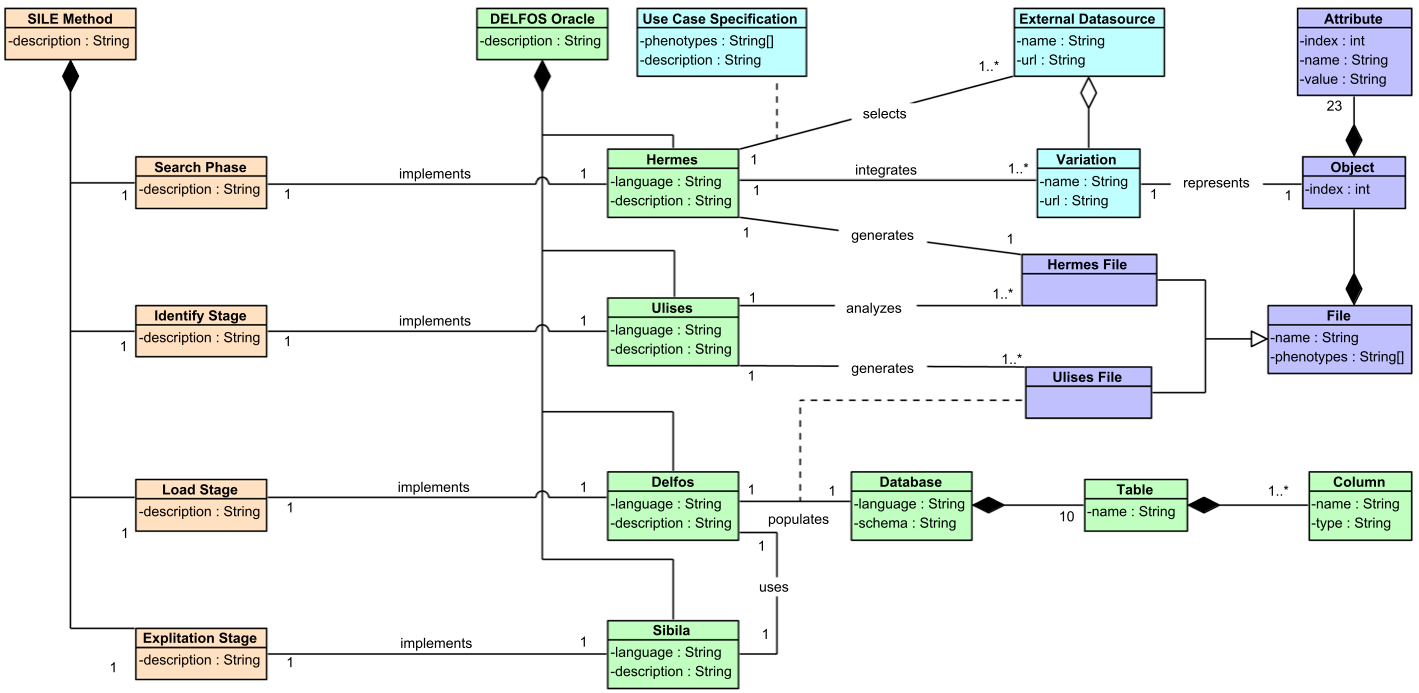
UML class diagram representing a simplified view of the components of the SILE method and the DELFOS oracle and how they are connected to each other. Orange: the fours phases of the SILE method. Green: the different elements that constitute the DELFOS oracle, each of which automates a specifix stage of SILE. Blue: the data external to the DELFOS Oracle that is used as input. Purple: intermediate files generated when executing the elements of DELFOS; they are temporary data that will be erased after executing the method

The DELFOS oracle consists of four independent but interconnected modules (Hermes, Ulises, Delfos, and Sibila) that implement each one of the stages of SILE (Search, Identification, Load, and Exploitation). The diagram in Fig. 4 shows the different components of our use case. The SILE method and its four phases are depicted in orange. The four software artifacts that instantiate the SILE method for a specific use case are depicted in green. The information and data that are external to the use case are depicted in blue (e.g., the variations that are analyzed by the DELFOS oracle). Finally, intermediate elements that are generated during the use of the DELFOS oracle (e.g., temporary data) are depicted in purple.

The use of the DELFOS oracle starts by selecting a phenotype or a set of phenotypes (Use Case Specification, Fig. 4) about which we want to determine what clinically relevant variations we can find in the different available genomic data sources. Then, with the Hermes module, we will be able to obtain the information available in the genomic data sources about the variations that are related to the selected use case. Next, with the Ulises module, we will process and analyze the information obtained with Hermes so we can identify the variations that are clinically relevant. Then, we will store all of the relevant variations and all their related information in Delfos. Finally, we will be able to exploit the stored information to create valuable clinical knowledge using the Sibila module.

The first module, Hermes, automatizes the Search stage of the SILE method. Hermes is an R package that performs the following tasks:

- Extract the information from a set of genomic data sources. Hermes implements specific connectors to four data sources (ClinVar^6^, Ensembl^7^, GWAS Catalog^8^, and LOVD^9^) that meet specific data quality criteria (believability, relevancy, reputation, currency, and accessibility) [7]. This task allows the information to be extracted in a simple and automatic way.
- Parse the information to comply with the CSSM. Hermes implements a parser for each selected data source, which transforms the different entities and concepts of the data extracted into the format defined by the CSSM. This task allows the data obtained in heterogeneous formats to be represented in a single standard defined by the CSSM. This facilitates the data analysis process performed in the other modules of the platform.
- Integrate the information. Hermes lets the user ingrate all of the data that is extracted and parsed in a single data structure, which facilitates the processing of the data by the other modules.

from the Hermes module, we obtain a file in a JSON format (from now on, *Hermes File*) that will store all of the integrated and parsed information about the genomic variations that is available on the selected databases. The Hermes File consists of different objects, each one of which represents a genomic variation (Variation). Each object contains a set of attributes or even other objects that represent different concepts of the CSSM.

The second module, Ulises, automates the Identification stage of the SILE method. This module is also an R package that implements an algorithm composed of a set of deterministic rules that filter and classify the genomic variations according to their clinical relevance. The rules are deterministic and each rule can only have two possible answers,“it is fullfilled” or “it is not fullfilled”. An example of a rule of the algorithm is assessing whether or not the variation is located in a gene with a demonstrated connection to the phenotype under study. There are only two possible answers to this assessment, that the variation is located in a relevant gene or that it is not.

The algorithm allows for the connection of the DELFOS oracle with Explainable Artificial Intelligence (XAI) [36], since all of the results provided by the algorithm come with solid and precise explanations for the decisions taken. Let us see an example to illustrate this. The variation *NC_000014.9:23415650:C:T*^10^ has been interpreted to be pathogenic for the *Hypertrophic Cardiomyopathy* phenotype. Since the variation has relevant clinical significance (i.e., it is reported to be pathogenic), and the interpretation that supports this assertion has been performed by an FDA-recognized database, the algorithm classifies the variation as being clinically relevant with a strong level of confidence. The algorithm outputs the reason for this classification in its results. In the example reported above, the algorithm indicates that the classified variant is pathogenic and the interpretation has been performed by a highly-reliable expert.

This module takes the data obtained with Hermes (Hermes File) as input, and provides a file in a JSON format (from now on, *Ulises File*) as output, which contains all of the variations together with their clinical classification according to the implemented algoritm.

The third module, Delfos, automates the Load stage of the SILE method. For this purpose, it is composed of two components: i) a MySQL database that instantiates the conceptual schema shown in Fig. 3, and ii) a web-interface that automates and facilitates the loading process. The technology used to store the data can be adapted to the user’s needs and the context of the research in order to deliver the most appropriate solution. This third module receives the file that has been obtained from the Ulises module (Ulises File), and it stores those variations classified as being relevant for the Ulises’ algorithm together with all of their related information.

Finally, the fourth module, Sibila, automatizes the Exploitation stage of the SILE method. Sibila is a web-based Genomic Information System (GIS) that connects with the database of the third module and gives the user mechanisms to exploit the information stored in it in order to generate useful and clinically relevant knowledge. The mechanisms for information exploitation are based on GenomIum visualization patterns [37] that are ontologically grounded in the CSG.

In its current state, the DELFOS oracle cannot deal with the different changes that may be found in the genomic information over time. It is a *static* platform in the sense that all of the new data substitutes the previous data, which hinders the analysis of the data evolution over time. The DELFOS Oracle is currently being used in multiple research projects in which we collaborate with clinical partners, as explained in the following subsection.

### A real-world need

Interpreting the clinical significance of variations is a long-lasting issue; yet, it continues to be one of the most severe bottlenecks that prevent achieving proper personalized medicine [38]. This is also the case of the research projects that we are currently working on, which has motivated this manuscript. In these research projects, CARDIOVAL (INBIO 2021/AP2021-05) and OGMIOS (INNEST/2021/57), we work with the following clinical partners: the Health Research Institute from the Hospital La Fe, València, Spain (IIS LA FE); the Foundation for the Research of the Hospital Clínico, València, Spain (INCLIVA); and the Foundation of the Valencian Community for the Management of the Institute for Health and Biomedical Research of the Hospital de Alicante, Alicante, Spain (ISABIAL). In these projects, the DELFOS oracle and the SILE method are being used to identify variations that are associated with pediatric oncology and familiar cardiopathies with risk of sudden death.

The different clinical experts involved in the projects have indicated that managing the temporal dimension was crucial in achieving their goals. They shared with us their worries regarding how to deal with the current situation, where the changes in the genomic information have a deep impact on their work and pose the need for changing past, present, and future clinical actions. Incorporating the mechanisms to deal with the changes of the genomic data over time in the SILE method and the DELFOS oracle is vital in the context of these projects.

Our objective is twofold. The first objective is to base our proposed solution in a well-established frame of knowledge by determining which genomic concepts change over time, why they change, and how to deal with these changes. The second objective is to design a solution to manage the temporal dimension in the context of the SILE method. In previous work [39], we explored a first, preliminary approach on how to achieve the goals herein presented. This manuscript continues these efforts. Ultimately, being able to manage the evolution of genomic data over time will allow us to provide physicians and clinicians with real-time updates of the variations that are relevant to them.

## Methods

The objective of our research is to improve the management of genomic data regarding its high degree of variability over time in the context of the DELFOS oracle. We followed an adapted version of the Design Science (DS) methodology proposed by prof. RJ Wieringa [40]. This methodology proposes to solve problems by studying the interaction of artifacts in a given context. Our context of work is genomics and the identification of relevant variations and the artifacts are the CSSM and the DELFOS oracle.

In DS, the work is based around answering research questions. In our case, our research on is the following: *How can the DELFOS oracle platform manage the temporal dimension of genomic data?*

The Design Science methodology offers different solutions to answer research questions, from which we chose to adapt the design cycle solution. A design cycle has three activities: Problem Investigation, Treatment Design, and Treatment Validation. Problem Investigation consists of describing the problem context and getting the required knowledge to improve that problem. This was performed in the previous ''Background'' section. Treatment design consists of providing an artifact to solve the problem identified in the previous activity. We divided this phase into three tasks:

1. *Study the characteristics of each database from which the DELFOS oracle extracts data* in order to understand their strengths and limitations. At the time of writing this manuscript, the databases are ClinVar^11^, Ensembl^12^, GWAS Catalog^13^, and LOVD^14^. We must determined th frequency their data is updated and whether or not they provide a way to retrieve historical data.
2. *Characterize which genomics concepts of the CSSM change over time*. We have to identify the reasons behind each change in order to characterize it correctly and to be able to manage it.
3. *Update the DELFOS Oracle* so that its architecture can manage the temporal dimension from a practical perspective.

Finally, Treatment Validation consists of investigating the interaction of the designed treatment, i.e., an updated version of the Delfos oracle, in the context of interest, i.e., genomics. We carried out the validation with a use-case where we studied how the proposed treatment deals with the changes observed in genomic data associated with the Long QT Syndrome, a familiar cardiopathy disease. The analyzed data was obtained from ClinVar, one of the most commonly used genomic data repositories.

## Results

### Study the characteristics of each database from which the DELFOS oracle extracts data

The first step in characterizing the temporal dimension of the data is to study the characteristics of each selected data source in order to determine how to extract the information stored in them, and the frequency at which the information they store is updated. As we have mentioned above, the DELFOS oracle provides support to the ClinVar, Ensembl, GWAS Catalog, and LOVD databases, so these are the data sources that we analyze in this section.

ClinVar is an open repository that provides information about the relation between genomic variations and disease development or treatment response. It is supported by the National Center for Biotechnology Information (NCBI), and it is updated weekly. With regard to the data extraction process, the information is available through *E-utilities*, which is a set of programs that provides an interface access to the information.

Ensembl is a database that stores information about the genome of different vertebrate species. This information includes the relation between genomic variations and disease development which is obtained from other data sources such as ClinVar. It is supported by the European Bioinformatics Institute (EMBL-EBI), and it is updated every three months. For the data extraction process, all of the data is available through REST services.

GWAS Catalog is a database that stores information about the statistical relations between genomic variations and phenotypes obtained from GWAS studies. It is supported by the National Human Research Institute (NHGRI) and the EMBL-EBI, and it is updated every two weeks. For the data extraction process, all of the data is available through REST services.

Finally, LOVD is a software platform that enables users to store the information of genomic variations in an individual and standardized database. Thus, LOVD is not a single database but rather a set of databases that follow a standardized format of representing the information. It is supported by the Molecular Health GmbH company; However, due to this structure, each of the installations of LOVD can have its own update policy, and it is not possible to define an update frequency. For the data extraction process, the data of each installation of LOVD is accessible through a web-service.

Table 1 shows a summary of the update frequency and the mechanisms available for the data extraction in each one of the data sources.

**Table 1:** Data sources used by the SILE method are displayed

Database	Update frequency	Data eExtraction
ClinVar	1 per week	E-utilities
Ensembl	1 per 3 months	REST services
GWAS Catalog	1 per 2 weeks	REST services
LOVD	-	web service

The first column indicates their name. The second column indicates the frequency at which their data is updated. The third column indicates the mechanism they provide to extract their data

From the characteristics of the data sources described here, it is clear that there is a great heterogeneity in the update frequencies. This heterogeneity must be considered by the DELFOS oracle and the SILE method when incorporating the treatment of the temporal dimension of the genomic data because these differences will affect the frequency at which is it necessary to update the body knowledge of the DELFOS oracle. This adds an extra layer of complexity to the problem we are dealing with, and it is a clear example of the impact that the heterogeneity among databases has on how we process genomic information.

### Characterize which genomic concepts of the CSSM change over time

To study the variability of the genomic variation information over time (i.e., to address the temporal dimension), we analyzed the temporal evolution of the genomic information associated with two diseases, namely, Early Onset Alzheimer’s Disease and Cystic Fibrosis.

The results of the study allowed us to identify that the genomic data obtained from the different databases with the DELFOS oracle can change because of the three following reasons:

1. The addition of a new variation.
2. The addition of a new link between an existing variation and a phenotype.
3. The change of an existing link between an existing variation and a phenotype with new information.

Identifying these three reasons is sufficient to correctly represent all of the possible changes occurring in the context of this work. The deletion of a variation is not considered because its existence is out of doubt once it has been identified. However, its level of evidence can change over time, either by increasing or decreasing the quality of the assertion criteria (even changing the variation pathogenicity). These changes in the information associated with variations are captured in the second and third items presented above.

Although these events may not seem enough to represent the complexity of the working domain, they are sufficient to cover our objectives. In the case of deletion, if a variation exists, it will not be removed; what could be removed is the link between that variation and a phenotype, which is considered in reason 3. We have covered this fact in the current version of the manuscript.

Below, we provide a detailed description of these three types of changes. For each change, we present a description of the entities of the CSSM affected that are by each change and exactly how they are affected (i.e., if there is a creation, a deletion, or update event affecting the instance of that particular entity).

The first reason is the addition of a new variation, which occurs when a new variation has been identified as being relevant for a given phenotype thanks to new scientific knowledge. For example, the *NC_000017.11:7674961:G:A*^15^ variation was first created in the ClinVar database in 2019. Therefore, at that time, we would have obtained a new variation in the JSON file provided by the Hermes module. To include a new variation into the DELFOS database, some instantiations of the entities present in the CSSM may have to change, as Table 2 shows.

**Table 2:** Actions that can be triggered on each entity of the model when a new variation is included in the database

Entity	Create action	Delete action	Update action
Actionability	Yes (Mandatory)	No	No
Bibliography	Yes (Optional)	No	No
ExternalItem	Yes (Mandatory)	No	No
Gene	Yes (Optional)	No	No
HGVSExpression	Yes (Optional)	No	No
Phenotype	Yes (Optional)	No	No
Significance	Yes (Mandatory)	No	No
Variation	Yes (Mandatory)	No	No
VariationPosition	Yes (Optional)	No	No

Explaining this in further detail, there are five entities of the CSSM that require creating an additional instance:

- *Significance* Only genetic variations that have a reported significance regarding a specific phenotype are stored in Sibila; thus, at least one significance will be created.
- *Actionability* Actionability, which is computed from the available clinical significances, is always created when incorporating a new variation.
- *ExternalItem* The new variation must be located in at least one external database.
- *Variation* The variation itself must be added to the database.

Besides, there are four entities of the CSSM whose instantiation is optional:

- *Bibliography* There are variations that have no associated bibliography.
- *Gene* If the gene that is affected by the variation already exists in the database, no changes are needed.
- *HGVSExpression* There are variations that do not have any associated HGVS expressions.
- *Phenotype* If the phenotype that is affected by the variation already exists in the database, no changes are needed.
- *VariationPosition* There are variations whose location in the DNA is unknown.

None of the entities will be affected by a delete or update action on an instance in this particular case.

The second reason is the *addition of a new link between an existing variation and a phenotype*. This change is triggered when a new link between a variation that already exists in the database and a phenotype is detected in the JSON file provided by the Hermes module. For instance, the *NC_000017.11:7674961:G:A* variation was updated twice in 2021, with the addition of a new link between the variation and a phenotype on each update. To add a new link between an existing variation and a phenotype in the database, some instantiations of the entities of the CSSM may have to change, as Table 3 shows.

**Table 3:** Actions that can be triggered on each entity of the model when a new link between an existing variation and a phenotype is included in the database

Entity	Create action	Delete action	Update action
Actionability	Yes (Mandatory)	No	No
Bibliography	Yes (Optional)	No	No
ExternalItem	Yes (Optional)	Yes (Optional)	Yes (Optional)
Gene	Yes (Optional)	No	No
HGVSExpression	Yes (Optional)	Yes (Optional)	No
Phenotype	Yes (Optional)	No	No
Significance	Yes (Mandatory)	No	No
Variation	No	No	Yes (Optional)
VariationPosition	Yes (Optional)	No	No

Specifically, there are two entities that require creating an additional instance:

- *Significance* Just like in Table 2, at least one significance will be created when a new link between a variation and a phenotype is established.
- *Actionability* If a new link between the existing variation and a phenotype is added, then at least one new clinical significance is created; thus, the actionability must be created or updated. The actionability will be created if there are no other links between the variation and the phenotype (i.e., it is the first link). The actionabilty will be recalculated if there are other links between the variation and the phenotype (i.e., other links existed before).

Besides, there are four entities whose instances may optionally change (i.e,, be created, updated, or deleted):

- *Bibliography* New bibliography may be generated to justify the new link between the variation and the phenotype.
- *ExternalItem* Additional databases containing an existing variation may be included over time, and previous databases can be deleted. Besides, the identifier or URL of a variation in a database can also change over time.
- *Gene* Overlapping genes exist in the genome. An existing variation can be located in a newly discovered gene (which overlaps an existing one) that is of interest for a phenotype. In this situation, the new gene must be created.
- *HGVSExpression* The HGVS standard allows for creating multiple expressions for the same variation, but an inappropriate use of the standard can lead to incorrect expressions. Consequently, new HGVS expressions can appear while others can be deleted.
- *Phenotype* If the new phenotype does not exist, it is included in the database.
- *Variation* Some aspects of the existing variation can be updated such as the variation’s type, the alleles, or any of the attributes described in the variation’s entity (see ''The ISGE method and the conceptual schema of the SILE method'' section) .
- *VariationPosition* Over time, new assemblies can be generated and included as a new position of a variation. Besides, a previous variation with unknown position in the DNA sequence can be properly identified and located.

The third reason is the *change of an existing link between a variation and a phenotype with new information*, which is triggered when some aspect or quality of a link between a variation and a phenotype is modified in the JSON file obtained with the Hermes module. For instance, an example of a change in an existing link is when a new gene is discovered in the location of an existing variation. Table 4 shows the possible changes in the instantiations of each entity in order to update a link between a variation and a phenotype.

**Table 4:** Actions that can be triggered on each entity of the model when an existing link between a variation and a phenotype is updated in the database

Entity	Create action	Delete action	Update action
Actionability	No	No	Yes (Optional)
Bibliography	Yes (Optional)	No	No
ExternalItem	Yes (Optional)	Yes (Optional)	Yes (Optional)
Gene	Yes (Optional)	No	No
HGVSExpression	Yes (Optional)	Yes (Optional)	No
Phenotype	Yes (Optional)	No	No
Significance	Yes (Optional)	Yes (Optional)	Yes (Optional)
Variation	No	No	Yes (Optional)
VariationPosition	Yes (Optional)	No	No

In this case, there are no entities whose instantiation is mandatory, but the instantiations of some entities may optionally change:

- *Actionability* The actionability of a variation for a phenotype can change if the set of significances used to compute its value changes.
- *Bibliography* New bibliography can be generated to represent new knowledge in the domain regarding an existing link between a variation and a phenotype.
- *ExternalItem* Additional databases containing an existing variation can be included over time. Previously selected databases can be deleted for multiple reasons. Besides, the identifier or URL of a variation in a database can also change over time.
- *Gene* The role of genes in the expression of phenotypes is an ever-changing matter of study. A gene that was not identified as a gene of interest for a phenotype can change this qualification. The gene that becomes of interest for a phenotype can contain an existing variation. In this situation, the new gene must be created.
- *HGVSExpression* New HGVS expressions can appear, and others can be deleted.
- *Phenotype* The phenotype cannot be modified because it already exists in the database.
- *Significance* The set of significances can change over time, and these changes must be represented in the database, e.g., the method or criteria used to obtain that significance.
- *Variation* Some aspects of the existing variation can be updated, as described in the previous case.
- *VariationPosition* Over time, new assemblies can be generated and included as a new position of a variation. Besides, a previous variation with unknown position in the DNA sequence can be properly identified and located.

**Table 5:** Matrix showing the costs associated with the temporal dimension in the context of the SILE method

Entity		Change		
		New variation	New link	Update link
Actionability		1	1	[0,1]
Bibliography		[0,1]	[0,1]	[0,1]
ExternalItem		1	[0,1] + [0,1] + [0,1]	[0,1] + [0,1] + [0,1]
Gene		[0,1]	[0,1]	[0,1]
HGVSExpression		[0,1]	[0,1] + [0,1]	[0,1] + [0,1]
Phenotype		[0,1]	[0,1]	[0,1]
Significance		1	1	[0,1] + [0,1] + [0,1]
Variation		1	[0,1]	[0,1]
VariationPosition		[0,1]	[0,1]	[0,1]
Change cost	Best	4	2	0*
	Worst	9	12	14

The table indicates the cost (best and worst cases) of the actions triggered by a specific change. For instance, the cost associated with a new variation ranges between 4 and 9 units of time.$*$ Although the theoretical cost of the best case is 0, the practical cost of the best case is 1

To estimate the computational cost of updating the database to incorporate the data changes that appear over time, we have calculated the computational cost in the best and worst scenario of creating a new variation, creating a new link variation-phenotype, or updating an existing link in the database. The results are shown in Table 5. In the best-case scenario, where only the mandatory instantiations are created/updated/deleted, creating new variations will have a higher impact when compared to the other two changes. This is because there is no previous information in the database. In the worst-case scenario, a change has affected the maximum possible number of instances. Adding a new link to an existing variation or updating it has a much lower impact in the best case because most of the information already exists in the database; however, the worst-case involves updating more information, with the extra cost that is associated with such operations.

### Update the DELFOS oracle

**Fig. 5:**
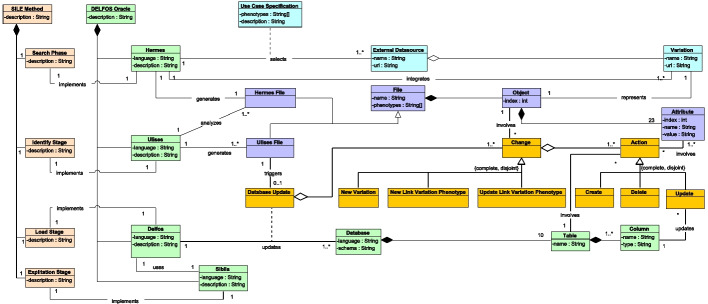
Updated UML class diagram depicted in Fig. 4.The new classes added to support the temporal dimension are depicted in dark orange. These classes represent database updates that are generated each time the Delfos Oracle is updated. Each database is composed of a set of changes, and each change is composed of a set of actions

In this section, we report the changes proposed in the DELFOS oracle to deal with the temporal dimension. Figure 5 shows these changes, which are limited to the load and exploitation stages of the SILE method (i.e., Delfos and Sibila). On the one hand, Delfos must be able to update the data of its database. On the other hand, Sibila must be able to display these changes intuitively.

#### The load phase

In the previous version of the DELFOS oracle, the JSON file was used to populate the database. This action considered that there was no previous data related to this Ulises File in the database. In the new version, the generation of a Ulises File might trigger a “database update” if there is new information in the Ulises File. A “database update” consists of at least one change, which is one of the three possibilities defined in ''Study the characteristics of each database from which the DELFOS oracle extracts data'' section, namely, the addition of a new variation, the addition of a new link between a variation and a phenotype, or the update of an existing link between a variation and a phenotype. Each change is composed of a set of actions, which are reported in Tables 2, 3, and 4. Every action will involve at least one attribute and exactly one table of the database. For instance, the “create” action in the Variation table of the database involves seven attributes, one per column of the table. The action is linked to a column if it consists of an update. This means that updating four attributes of a given table will be represented with four “update” actions.

For each phenotype in the database, the SILE method is executed every week (according to Table 1, this is the lowest update frequency value), and we have implemented a mechanism to check whether the database should be updated or not using the generated Ulises File as input. If a “database update” is triggered, a set of algorithms that perform the update are executed. Formula 1 shows a simplification of this algorithms.
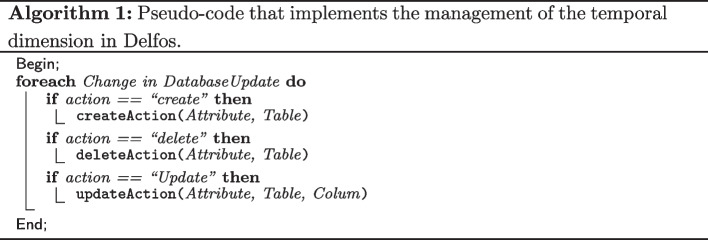


#### The exploitation phase

Once we have defined the mechanisms to update the database when new data is generated in the external data sources, we must define how to display this new genomics information in Sibila. A correct visualization of this information facilitates the analysis of the changes in the information over time. Incorporating such a critical dimension in the exploitation of genomic data has relevant consequences in clinical practice since it allows clinicians to update the genetic reports of their patients with the most up-to-date data.

We conducted a set of interviews with domain experts to reveal their actual needs in terms of the temporal dimension. The collected feedback has been grouped into three items, which have been incorporated into the Sibila platform:

- The first item is related to being able to inspect the database considering snapshots on different dates. In essence, the user wants to work with a specific snapshot of the database. The set of data that is displayed in Sibila will depend on the snapshot selected by the user.
- The second item is related to being able to inspect the changes of variations and their associated data over time. The user needs to track the changes in the data of a specific variation, e.g., the bibliography that is relevant for a variation or the set of genes that the variation affects. This item is relevant in order to identify when new relationships with a phenotype appear or what specific changes arise when existing relationships are updated.
- The third item is related to being able to inspect the changes of phenotypes and their associated data over time. Users need to track the changes in the data of a specific phenotype. We want to give them a mean to analyze when new variations related to the phenotype and what changes in the relationship with a previously reported phenotype appear.

To address the temporal dimension in Sibila, we must provide solutions for all three of these use cases. We have followed a pattern-based approach, which consists of creating/updating UIs by composing visualization patterns. A well-designed UI allows users to perform their tasks efficiently and facilitates the achievement of their goals.

**Fig. 6:**
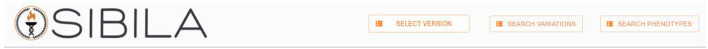
Top var of Sibila with a new button to select the Delfos database version

**Fig. 7:**
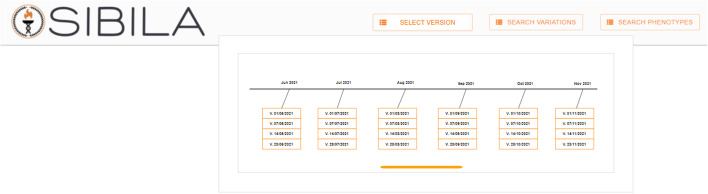
Top bar of Sibila with the drop-down that appears after clicking the SELECT VERSION button

For the first item, in the top bar of the Sibila web-page, we incorporated the option to select the version of Delfos that the user wants to work with. The Delfos database is updated every week. The user can select which version to use by clicking on the *SELECT VERSION* button (see Fig. 6) and by selecting the desired version of the Delfos database in the drop-down window that is shown (see Fig. 7).

**Fig. 8:**
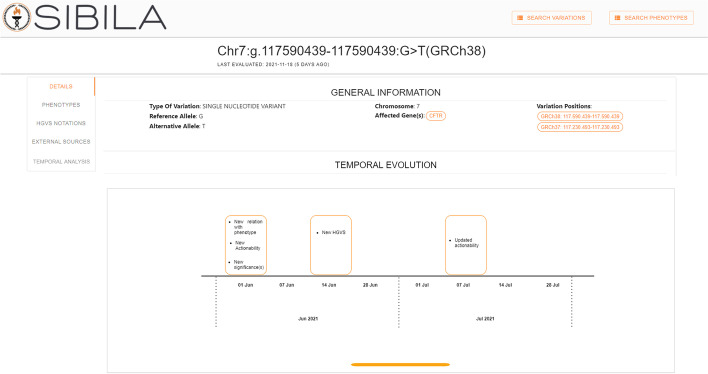
Subsection TEMPORAL EVOLUTION on the variation’s main page

For the second item, Sibila must allow the user to inspect the changes that occur on the variation information over time. For this purpose, we have introduced a set of modifications in the Sibila variation section. First, on the main page of each variation (*DETAILS* section), we have introduced a new subsection called *TEMPORAL EVOLUTION* that provides a summary of all of the changes regarding the variation data. Specifically, this section displays a temporal line where all of the changes that have been detected over time are represented, at the precise moment they are detected. Figure 8 shows the updated section.

**Fig. 9:**
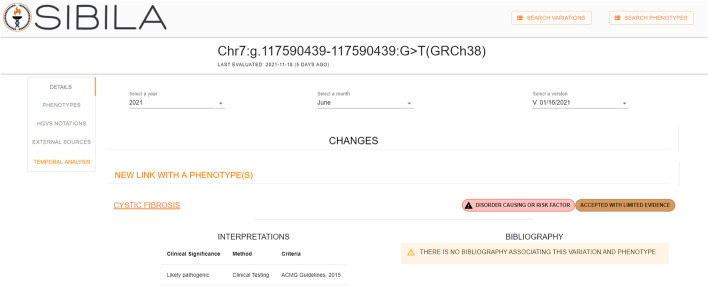
Section TEMPORAL ANALYSIS on the variation page

The *TEMPORAL EVOLUTION* subsection presents a useful summary of the changes in a variation’s data over time, but the user may be interested in knowing exactly what data has changed. For instance, the *TEMPORAL EVOLUTION* subsection will indicate that a new bibliography item has been introduced, but the specific information about this new item will not be provided. Therefore, to provide the user with more precise information about changes that occur over time, we have included a new section in the variations page called *TEMPORAL ANALYSIS*, which is displayed in Fig. 9. At the top of this section, three drop-down menus will help the user to navigate among the different snapshots of the Delfos database. The first two drop-down menus will allow the year and the month to be selected to filter all of the snapshots of the Delfos database that are available. In the third drop-down, the user will select the specific snapshot to be consulted. Once the snapshot is selected, detailed information about the identified changes will be displayed in a subsection called *CHANGES*. For instance, if a new bibliographic item was detected in the selected snapshot, information about the title, author, and year will be displayed. Therefore, the *TEMPORAL ANALYSIS* section will provide the user with the necessary tools to perform a thorough analysis about the temporal evolution of genomic information over time.

For the third item, Sibila must allow the user to inspect the changes that occur over time in the data associated with a phenotype. In terms of information needs, the case of the phenotypes is very similar to the case of the variations. For this reason, and to give coherence to the interface of Sibila, the same solution described for the section of the variations has been implemented for the section of the phenotypes. Therefore, on the main page of each phenotype (*DETAILS* section) we have introduced a new subsection called *TEMPORAL EVOLUTION*. It locates the changes found in the data of a phenotype on a temporal line at the precise moment that they have been found. Finally, to provide the user with a more thorough analysis of the temporal dimension, a new section called *TEMPORAL ANALYSIS* has been introduced. This section will have the same structure described in the case of the variation section and, therefore, will allow the user to perform a thorough analysis of the temporal dimension.

## Use case: the long QT syndrome

We have tested our approach by using the improved version of the DELFOS Oracle for Long QT Syndrome data. The Long QT Syndrome is a familiar cardiopathy that is associated with the risk of sudden death, and it is currently under study in two research projects in which we are involved.

For this purpose, we gathered all of the data that is available for the Long QT Syndrome from January 2020 to November 2021 in ClinVar, as it is the only source that provides historical data in an accessible manner. To perform the testing, we followed the steps described below:

1. We downloaded the whole ClinVar database for the months from January 2020 to November 2021. We obtained a processable file for each of the months selected. All of the data is available in the FTP service of ClinVar^16^.
2. We filtered each of the files so that only the variations associated with the Long QT Syndrome are present. Then, we transformed each filtered file to a format that is compliant with the CSSM by using the parser of the Hermes module, obtaining a Hermes File for each of the months.
3. Each of the Hermes Files was passed to the Ulises module, obtaining the corresponding Ulises Files with the variations associated with the Long QT Syndrome classified according to their relevance.
4. Starting from the empty Delfos database, we uploaded each of the files to the database and analyzed the database updates that were triggered. Specifically, we analyzed how the data changed each month by considering three scenarios: the number of reported variations, the number of new associations between variations and phenotypes, and the number of updated associations between variations and phenotypes.

The results of the specified testing helped us determine the practical utility of the improvements introduced in the DELFOS Oracle.

**Fig. 10:**
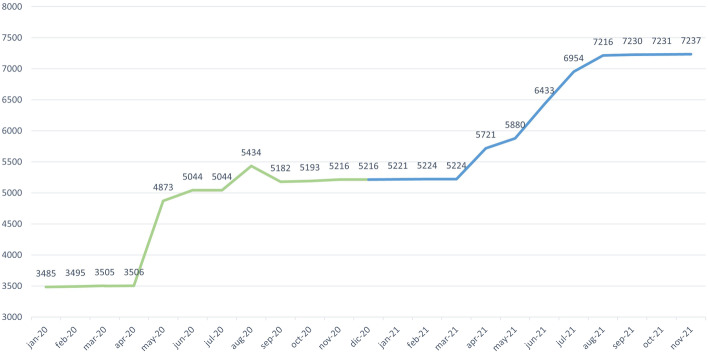
Number of variations per month

With regard to the first of the analyzed scenarios (i.e., the evolution in the number of reported variations), Fig. 10 shows the number of variations associated with the Long QT Syndrome found in each of the months studied. The number of variations found increased continuously, which is in line with the constant growth of genomic data; However, there was an exception between August 2020 and September 2020. In August 2020, we found 5434 variations, whereas this number decreased to 5182 in September 2020. According to ClinVar, the new variations reported in August 2020 were probably deleted by their curators in September 2020 when reevaluating them. This example clearly shows the importance of ensuring the presence of suitable mechanisms that allow users to keep track of the temporal evolution of genomic data.

**Fig. 11:**
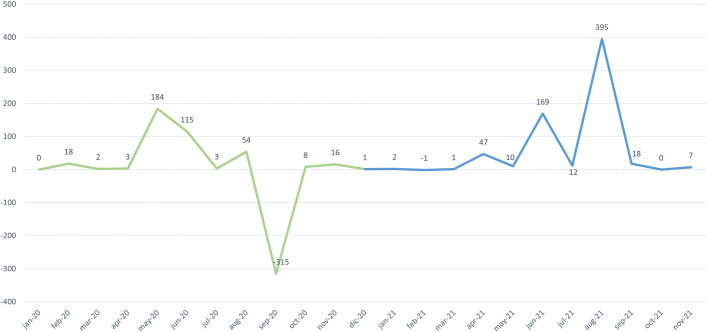
Number of new variation-phenotype links per month

With regard to the second of the analyzed scenarios (i.e., the evolution in the the number of new associations between variations and phenotypes), Fig. 11 shows the number of new associations between existing variations and a phenotype in each of the months studied. Unlike the first scenario, there was a high degree of variability in the number of new associations obtained each month, without a clear tendency. There were two months, namely, September 2020 and February 2021, where the number of new associations decreased. The variations that were removed each month to be reevaluated (see the first scenario) involved removing their associations to every phenotype. This is the reason for the decrease in associations between variations and phenotypes in September 2020 and February 2021. There are months such as May 2020, June 2021, and August 2021 that had a significant increase in the number of new associations, which is a clear indicator of the considerable variability of genomic data.

**Fig. 12:**
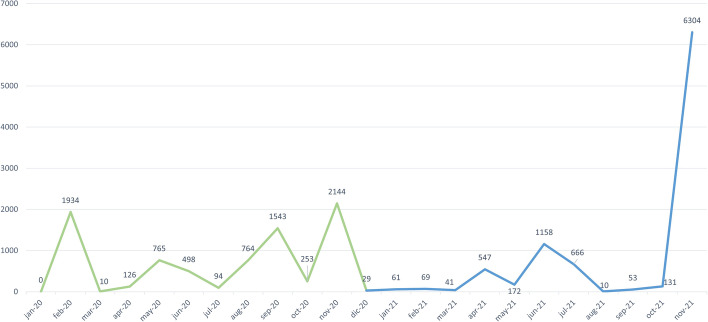
Number of updated variation-phenotype links per month

Finally, Fig. 12 shows the number of associations updated each month regarding the third of the analyzed scenarios. As in the previous case, there was very high variability in the number of updated links per month. However, it was surprising that there were months such as February 2020, September 2020, November 2020, June 2021, or November 2021, where more than one thousand links were updated. This might be due to the high degree of variability of genomic data. We provide a more detailed explanation below, discussing the relevance of these changes in detail.

We studied how many of the associations that were updated each month for the third scenario were relevant. We consider that an update is relevant if it modifies the clinical relevance of the variation for a phenotype or if it changes an essential aspect of the variation. Considering the conceptual schema represented in Figure 3, the changes that affected the the following classes were defined as being relevant:

- *Bibliography* The literature is essential to support the reported relationship between a variation and phenotype. The Ulises algorithm uses the literature associated with variations to classify them, and any change is expected to affect the results provided.
- *Gene* Changes in the gene affected by the variation (e.g., the addition of a new gene) may affect the results provided by the Ulises algorithm because it analyzes the relevance of the gene regarding the phenotype under study. The fact that there is a new gene that is relevant to the phenotype associated to the variation can imply a change in the clinical relevance of the variation.
- *VariationPosition* Changes in the location of the variation are essential to be able to analyze the variation. This change includes identifying the variation’s location for the first time or including its position in a new assembly.
- *Significance* The significance of a variation determines the importance of the relationship between a variation and a phenotype. Thus, any change in significance (update, addition, or deletion) has an important impact on the significance of the variation.
- *Variation* Changes in attributes of the variation (e.g., its type, its alleles) are required to characterize it; thus, they are essential to be able to study it.
- *Actionability* This represents the practical importance of a variation for a phenotype. It is a derived value that is obtained from the clinical significances reported by the curators and experts. Any change in the actionability of a variation for a phenotype will affect the clinical relevance of this variation.

**Fig. 13:**
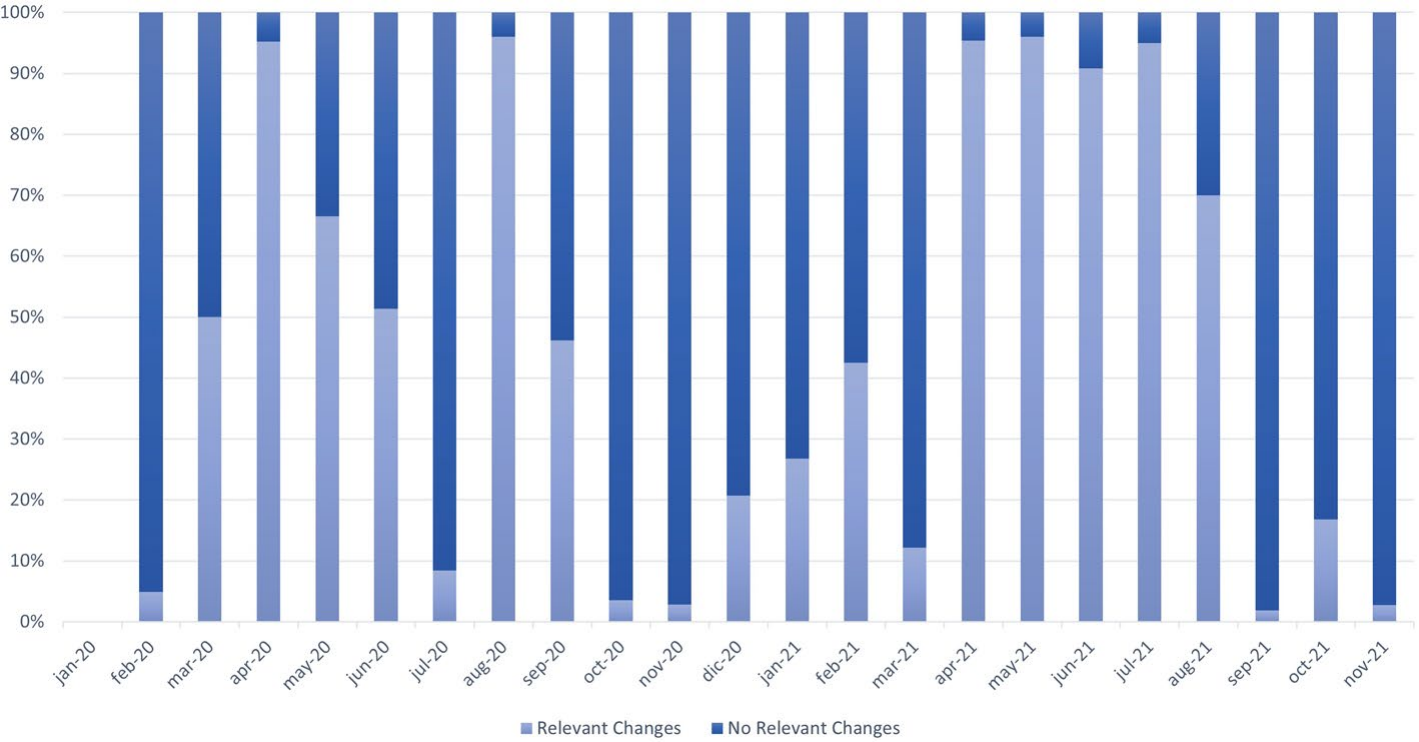
Percentage of relevant and non-relevant changes per month

Once we determined the changes that were considered to be relevant, we calculated the percentage of relevant and non-relevant changes in each month regarding the previous one. Figure 13 presents the results.

There was a high degree of variability in the percentage of relevant changes each month. While November 2021 only had 2.7% relevant changes, May 2021 had a 96%. We expect relevant consequences from a clinical perspective in those months when relevant changes are the majority. The situation reported above explains the importance of appropriately dealing with the temporal dimension of the genomic data. Table 6 represents the number of changes found in each of the classes described as relevant (Significance, Actionability, VariationPosition, Gene, Variation).

**Table 6:** Number of changes in each relevant class per month

Month	Significance	Actionability	VariationPosition	Gene	Variation
Jan-20	0	0	0	0	0
Feb-20	99	18	0	0	0
Mar-20	5	2	0	0	0
Apr-20	19	10	101	0	0
May-20	557	112	0	0	0
Jun-20	198	103	61	0	0
Jul-20	6	0	1	1	0
Aug-20	740	67	0	0	0
Sep-20	22	0	691	0	1
Oct-20	9	12	0	0	0
Nov-20	61	4	0	0	0
Dec-20	1	0	5	0	0
Jan-21	9	1	0	0	1
Feb-21	28	22	0	0	1
Mar-21	5	0	0	0	0
Apr-21	539	4	0	0	0
May-21	170	0	0	0	0
Jun-21	467	7	671	11	2
Jul-21	638	4	0	0	0
Aug-21	7	1	0	0	0
Sep-21	1	0	0	0	0
Oct-21	22	2	0	0	0
Nov-21	17	4	0	0	0

Table 6 shows that most of the relevant changes changed the significance of variations. This insight means that new interpretations associating variations and phenotypes appear over time. In addition, these new interpretations can change the variation’s actionability and the quality of the data supporting that actionability. We found that these changes tend to make variations relevant.

Some months had a high number of changes regarding the location of variations, especially in April 2020, September 2020, and June 2021, where this type of change was the most common. Usually, these changes can: i) provide a position that was previously unreported; ii) add a position using another assembly; or iii) modify a previously reported position. Having variations that change their location over time directly impact the results of clinical analyses, which means that the number of pathogenic variations identified changes depending on when the test was performed.

Changes regarding genes and variations are much less frequent. All of the changes found in genes consisted of associating a variation with a new gene. New genes are identified over time, and their role in specific diseases can impact the evaluation of a variation’s pathogenicity. We found that most changes in variations are related to their type and alleles. In the case of type, there was a single reclassification from insertion/deletion to microsatellite. In the case of the alleles, some variations had their alleles reported over time.

**Fig. 14:**
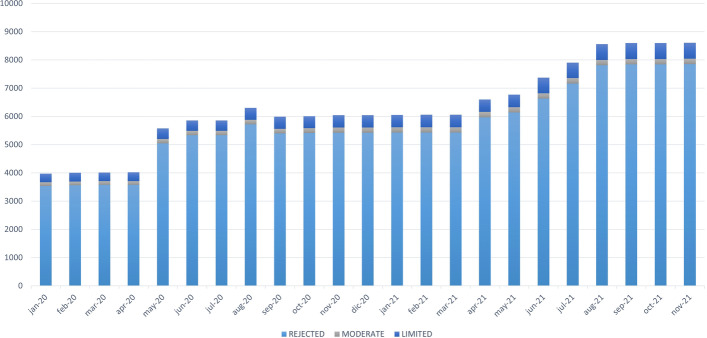
Classification of the variations per month

Finally, we studied how these changes affect results of the the Ulises AI algorithm. Figure 14 shows the evolution of the algorithm results over time for the Long QT Syndrome.

It can be observed that the number of classifications provided by the algorithm increased over time since the number of variation-phenotype associations increased each month. Most of the analyzed variations were rejected, followed by Accepted with Limited Evidence, and Accepted with Moderate Evidence. None of the variations analyzed in the defined period (i.e., 2020 and 2021) were classified as Accepted with Strong Evidence. Two factors cause the increase in the number of accepted variations over time. The first factor is the addition of new variations with a sufficient level of supporting information. The second factor is the addition of new supporting information of existing variations. However, most new data will be rejected due to insufficient supporting information. This tendency must be addressed due to the importance of this field and its direct impact on human health. This analysis demonstrates that genomic data changes continuously, and the clinical significance of variations can change from month to month. These insights constitute a clear indicator of the importance of dealing with the temporal dimension of genomic data.

## Discussion

Our study of the characteristics of each database from which the DELFOS oracle extracts data has shown that there is no clear tendency in their update frequency since they range between once per week to once every two weeks (see Section 3.1). Therefore, the selection of an appropriate update frequency is left to each data source administrator. However, it is clear that updates should be done at a constant and frequent rate since our study has shown that the amount of data that is being updated increases every year.

The case of LOVD is a special one because its architecture is based on several independent databases. As a result, there is no standard update policy, and, while some databases were updated in 2021, others were updated in 2012. Even worse, the update time of some databases is unknown.

We believe that our community would benefit from achieving data sources with more frequent updates. However, defining a standardized frequency for updating the data would be an appropriate and necessary starting point. The absence of this frequency poses a threat to the generalization and scalability of our approach. We will have to study the updating strategy of each database added instead of considering a single frequency, which increases the time and effort needed to manage the temporal dimension.

An example of the high heterogeneity in how the temporal dimension is treated in the data sources is that ClinVar is the only one that provides historical data in an accessible manner. This situation represents the main limitation in the performed use case (see Section 4) because we can only report and analyze the changes in Clinvar, not in the rest of the datasources.

With regard to the characterization of changes in genomic concepts over time, there are three reasons behind a data change (see ''Characterize which genomic concepts of the CSSM change over time'' section). An analysis of the results of ''Use case: the long QT syndrome'' section, shows that the number of relevant changes (i.e., those changes that modify the clinical relevance or an essential aspect of a variation) differs dramatically from month to month (see Fig. 13). This result reinforces that a constant tracking of genomic data is necessary in order to deliver the best possible outcome in precision medicine. In approximately 27% of the total number of months studied, almost 100% of the changes detected were relevant. In the other months, there was not a clear tendency regarding relevance/non-relevance.

**Fig. 15:**
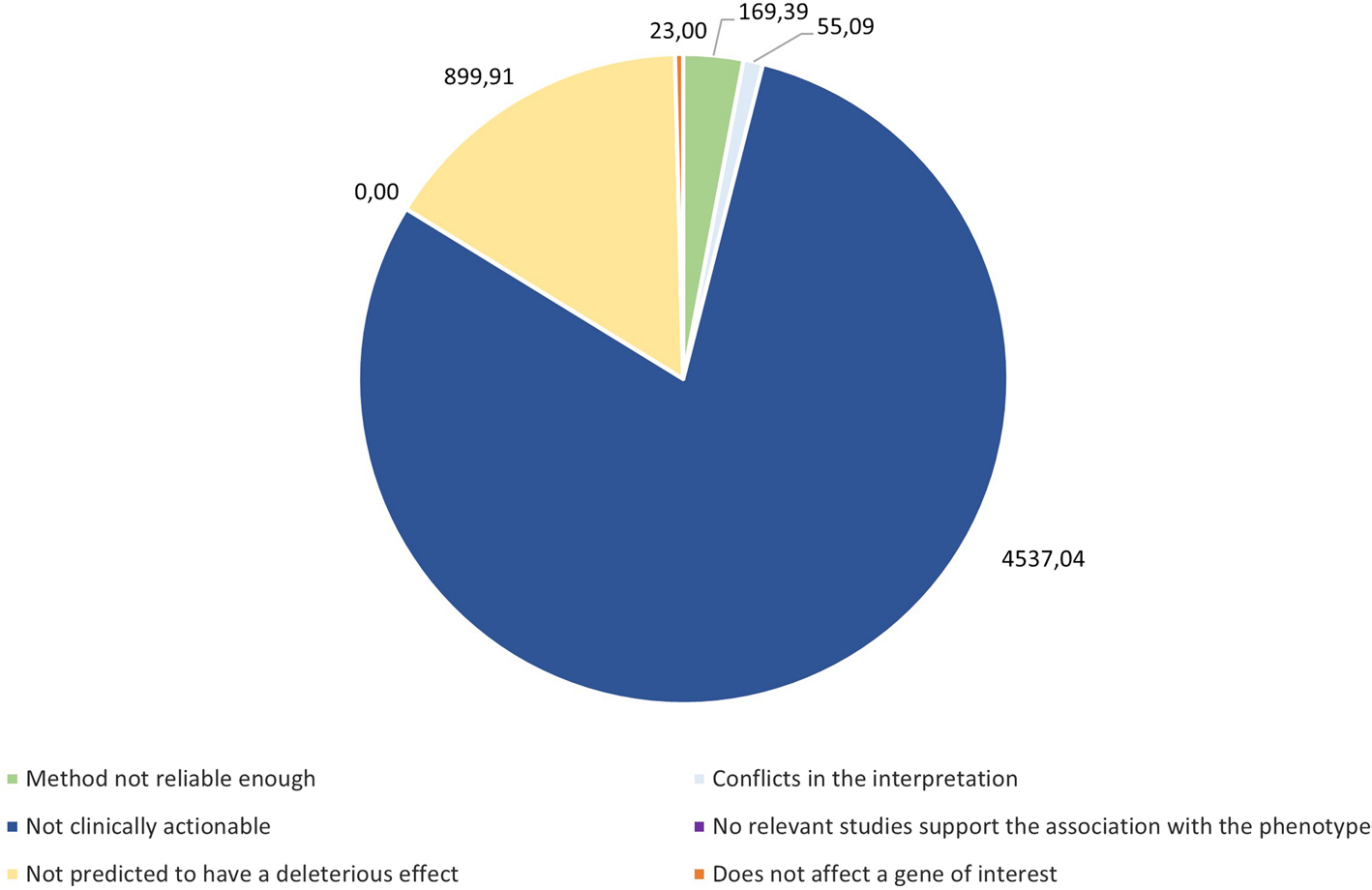
Average number of rejected variations per reason

The rate of accepted variations was constant throughout the analysis, meaning that most variations are rejected. As Fig. 14 shows, less than 10% of the analyzed variations were accepted by the DELFOS oracle. The reasons for these rejections are shown in Fig. 15. Almost 80% of the analyzed variations were rejected because they were not clinically actionable, which means that they were associated with a benign or an unknown effect, and were therefore not of interest in this use case. The second most common reason for rejection is because variations are not predicted to have a deleterious effect, which means that they have not been interpreted (i.e., they do not have a clinical significance) and the predictors, such as SIFT^17^ or Polyphen^18^, do not consider them to be deleterious. Although they are not the majority (an average of 23 variations), having variations rejected because of their conflicting interpretations is a significant indicator of the need to increase the use of standards and guidelines, such as the ACMG/AMP [19] for classifying variations.

Finally, the update of the DELFOS oracle allowed us to implement and handle the temporal dimension (i.e., how genomic data that is relevant to clinical diagnosis changes over time). This evolution allowed us to change from a *static-data* perspective to a *dynamic-data* perspective. This means that the data stored at the DELFOS oracle evolves over time, which will improve the outcome of genetic diagnosis performed in the future as well as updating and correcting past genetic diagnoses. However, expanding the platform comes with a trade-off of complexity, especially in the module in charge of the Load phase, which now has to update the data in addition to adding it.

Based on the analysis of the selected databases and the changes that can occur in their data, we updated the DELFOS oracle platform. The new version can manage the temporal dimension by means of identifying and storing the changes produced in the entities and their attributes each time the DELFOS oracle database is updated. These changes are consolidated in what we have called a *database update*, which constitutes the detailed set of changes between two different versions. Apart from tracking the changes in genomic data, we can deliver state-of-the-art medical reports and carry out studies regarding the temporal evolution of data such as the one presented in the use case of this manuscript.

The approach herein presented is well-designed and allows us to manage the temporal dimension of genomic data. The main limitation is the fact that changes in the data are not captured until we execute a database update. This situation means that if new databases that update more frequently are included, we will have to reevaluate the frequency at which we perform a database update.

## Conclusions

We have seen that characterizing the temporal dimension is a complicated task in genomics because of the inherent data complexity and heterogeneity. However, efficiently managing this dimension impacts patients’ diagnosis and treatment because it will be the first step to allow moving from a static genetic report to a dynamic one that improves over time. This justifies the relevance of this work, where we have proposed an approach for a correct management of the temporal dimension in genomic data. This approach has been tested in a specific use case to show the high degree of variability in this domain and its relevance in clinical diagnosis.

Following this new approach, our work is structured in four steps: i) a study of the characteristics of each database from which the DELFOS oracle extracts data; ii) a characterization of the genomics concepts of the CSSM that change over time; iii) an update of the DELFOS oracle, and iv) a use case to validate these changes.

A study of the temporal variability of external data sources has been performed in the first step. As mentioned in ''The SILE method'' section, the SILE method selects data sources based on defined criteria. We plan to add additional requirements to these criteria in order to deal with the temporal dimension. Consequently, the cost of analyzing and including new data sources could increase, and some data sources might not be included depending on how they manage the temporal dimension.

In the second step, we characterized the three changes that can modify genomic data over time in our use case. The first change is the addition of a new variation; the second change is the addition of a new link between a variation and a phenotype; the third change is the change of an existing link between a variation and a phenotype. The first change is the one that enforces the higher number of entity changes because it forces us to create at least one new variation, new external item, new clinical significance, and new clinical actionability. With regard the computational cost of each change, the addition of a new variation action also has the highest cost in the best case but the lowest cost in the worst case. Changing an existing link between a variation and a phenotype is the action with the highest cost in the worst case since many different entities can be created, updated, or deleted.

In the third step, we updated the DELFOS oracle using a conceptual model-based approach, which allowed us to correctly manage all of the changes occurring in genomic data over time. The update process has followed a top-down approach in which we updated the conceptual models that ground its architecture and processes, and then we updated the platform. Specifically, we updated the Delfos module to change from a *static-data* perspective to a *dynamic-data* perspective, and we updated the Sibila module to display the new information to the users. The updated DELFOS oracle offers clinical experts the most current information for present clinical care and past diagnoses and treatments that may need to be reviewed.

Finally, in the use case, which consisted of analyzing variations that are related to the long QT syndrome, we demonstrate the importance of taking into account the temporal variability of genomic data when diagnosing patients. It is essential to provide tools and mechanisms to automate the updating of the data and the notification of clinicians of those changes. To illustrate, the number of variations related to this phenotype doubled in one year. The DELFOS oracle will help the clinicians that we work with to deliver better diagnoses and treatments for past, present, and future patients.

## Data Availability

The datasets generated and/or analysed during the current study are available in the ClinVar repository, https://ftp.ncbi.nlm.nih.gov/pub/clinvar/xml/. We used datasets ranging from January 2020 to November 2021.

## Footnotes

1 We use the Canonical SPDI to identify the variation. For more details regarding this nomenclature see https://www.ncbi.nlm.nih.gov/variation/notation/.,

2 https://www.ncbi.nlm.nih.gov/clinvar/variation/12379/?new_evidence=false.

3 https://www.ncbi.nlm.nih.gov/clinvar/variation/35555/?new_evidence=false.

4 https://www.ga4gh.org/.

5 An HGVS expression uses the HGVS nomenclature to represent changes at the DNA, RNA, or amino acid level. It describes the change and its location.

6 https://www.ncbi.nlm.nih.gov/clinvar/.

7 https://www.ensembl.org/.

8 https://www.ebi.ac.uk/gwas/.

9 https://www.lovd.nl/.

10 https://www.ncbi.nlm.nih.gov/clinvar/variation/36642.

11 https://www.ncbi.nlm.nih.gov/clinvar/.

12 https://www.ensembl.org/index.html.

13 https://www.ebi.ac.uk/gwas/.

14 https://www.lovd.nl/.

15 https://www.ncbi.nlm.nih.gov/clinvar/variation/418517/?new_evidence=false.

16 https://ftp.ncbi.nlm.nih.gov/pub/clinvar/xml/.

17 https://sift.bii.a-star.edu.sg/.

18 http://genetics.bwh.harvard.edu/pph2/
